# Comprehensive, Continuous, and Vertical Measurements of Seawater Constituents with Triple-Field-of-View High-Spectral-Resolution Lidar

**DOI:** 10.34133/research.0201

**Published:** 2023-07-19

**Authors:** Kai Zhang, Yatong Chen, Hongkai Zhao, Zhongping Lee, Emmanuel Boss, Iwona Stachlewska, Davide Dionisi, Cédric Jamet, Paolo D. Girolamo, Aleksey Malinka, Chengchong Jiang, Hongda Wu, Lingyun Wu, Feitong Chen, Xiaolei Zhu, Nanchao Wang, Chuxiao Chen, Qun Liu, Lan Wu, Yudi Zhou, Weibiao Chen, Dong Liu

**Affiliations:** ^1^ Ningbo Research Institute, State Key Laboratory of Modern Optical Instrumentation, College of Optical Science and Engineering, Zhejiang University, Hangzhou 310027, China.; ^2^ ZJU-Hangzhou Global Scientific and Technological Innovation Center, Zhejiang University, Hangzhou 311200, China.; ^3^ Donghai Laboratory, Zhoushan 316021, China.; ^4^ State Key Lab of Marine Environmental Science, College of Ocean and Earth Sciences, Xiamen University, Xiamen 361102, China.; ^5^ School of Marine Sciences, University of Maine, Orono, ME 04469-5741, USA.; ^6^ Faculty of Physics, University of Warsaw, Warsaw 02093, Poland.; ^7^ Institute of Marine Sciences, Italian National Research Council, Rome 00133, Italy.; ^8^ Université du Littoral Côte d'Opale, CNRS, Univ. Lille, IRD, UMR 8187 - LOG - Laboratoire d'Océanologie et de Géosciences, Wimereux F-62930, France.; ^9^ Institute Scuola di Ingegneria, Università della Basilicata, Potenza 85100, Italy.; ^10^ Institute of Physics, National Academy of Sciences of Belarus, Minsk 220072, Belarus.; ^11^ Key Laboratory of Space Laser Communication and Detection Technology, Shanghai Institute of Optics and Fine Mechanics, Chinese Academy of Sciences, Shanghai 201800, China.; ^12^ Intelligent Optics & Photonics Research Center, Jiaxing Research Institute Zhejiang University, Jiaxing 314000, China.; ^13^ Jiaxing Key Laboratory of Photonic Sensing & Intelligent Imaging, Jiaxing 314000, China.

## Abstract

Measuring the characteristics of seawater constituent is in great demand for studies of marine ecosystems and biogeochemistry. However, existing techniques based on remote sensing or in situ samplings present various tradeoffs with regard to the diversity, synchronism, temporal-spatial resolution, and depth-resolved capacity of their data products. Here, we demonstrate a novel oceanic triple-field-of-view (FOV) high-spectral-resolution lidar (HSRL) with an iterative retrieval approach. This technique provides, for the first time, comprehensive, continuous, and vertical measurements of seawater absorption coefficient, scattering coefficient, and slope of particle size distribution, which are validated by simulations and field experiments. Furthermore, it depicts valuable application potentials in the accuracy improvement of seawater classification and the continuous estimation of depth-resolved particulate organic carbon export. The triple-FOV HSRL with high performance could greatly increase the knowledge of seawater constituents and promote the understanding of marine ecosystems and biogeochemistry.

## Introduction

Various processes that are critical for marine ecosystems and biogeochemistry are associated with seawater constituents [1,2]. Measuring optical properties and particle size distribution (PSD), which are external expressions of type, refractive index, geometric shape, structure, and biological and chemical features of seawater constituents, makes important contributions to increase our knowledge regarding planktonic growth-metabolic processes [3], particle aggregation and fragmentation [4], phytoplankton blooms [5], biological pumps [6], etc. Furthermore, the temporal and spatial variability of ambient light in the oceans can be quantified, which affects practical applications ranging from prey–predator relationships to coastal bathymetric mapping, as it is a fundamental driver of marine productivity and upper ocean heat budget [7,8]. Therefore, high-resolution and accurate measurement techniques are highly desired for monitoring optical properties and PSD of seawater constituents to study marine ecosystems and biogeochemistry.

Passive optical remote sensing techniques provide surface information in large areas but have little sensitivity to vertical distributions of seawater constituents [9]. In situ methods can provide accurate and depth-resolved data, but they are limited in the amount of spatial and temporal coverage due to unavoidable contact with seawater [10]. Lidar, an active optical remote sensing technique, can obtain continuous and vertical optical properties during both day and night time [11,12], which has been used to study plankton [13], primary productivity [14], etc. Hitherto, most oceanic lidars have limited information, mainly including the backscatter and lidar attenuation coefficients [15]. The lack of absorption and scattering coefficients, as well as PSD, limits the lidar applications in marine science, including carbon cycles and seawater classification. Thus, it remains a challenge in optical remote sensing and in situ techniques to meet the simultaneous measurement demands of characteristic types, underway observations, and vertical information.

To overcome this conundrum, lidar signals, which are coupled with absorption, scattering coefficients, and PSD through multiple scattering effect, should be further analyzed to obtain more information of seawater constituents [16]. Studies have shown that the lidar attenuation coefficient can be regarded as the beam attenuation coefficient at a narrow field of view (FOV) near zero and the diffuse attenuation coefficient (*K*_d_) at a wide FOV [17], which was then verified by field measurements [12]. Therefore, a multi-field-of-view (multi-FOV) lidar system can potentially obtain more information than current single-FOV lidar systems. Furthermore, the particulate volume scattering function is highly complicated in the backward direction, leading to the quantifying of the multiple scattering become an arduous task [18]. The molecular Brillouin scattering could solve this problem due to its isotropic backscattering [11]. It is shifted about 7 to 8 GHz to both sides relative to the central wavelength of the laser, which can be discriminated by the high-spectral-resolution lidar (HSRL) technique. Thus, the combination of multi-FOV and HSRL techniques could be beneficial to solving the above conundrum.

Here, we demonstrate an oceanic triple-FOV HSRL for comprehensive, continuous, and vertical measurements of seawater constituents. With the well-designed and well-calibrated triple-FOV HSRL system, desirable performance is achieved for continuous profiles of absorption (*a*) (532 nm), scattering (*b*) (532 nm), and the slope of PSD (*ξ*), which are highly consistent with Monte Carlo (MC) simulations and in situ measurements. The system has been deployed in the South China Sea to measure the vertical structure and horizontal gradient of nearshore to offshore seawaters. Furthermore, potential applications in the seawater classification and estimating the particulate organic carbon export are presented, which have broad implications for understanding seawater constituents and related marine science studies.

## Results

### Triple-FOV HSRL technique

**Fig. 1.**
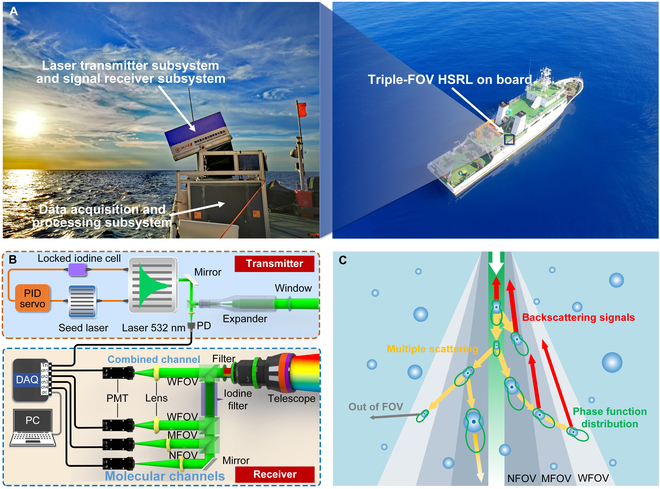
Principle of the shipborne triple-FOV HSRL. (A) System deployment geometry. (B) Schematic diagram of the triple-FOV HSRL system. WFOV, MFOV, and NFOV are for the wide FOV (200 mrad), middle FOV (80 mrad), and narrow FOV (40 mrad), respectively. (C) Simplified scheme for the triple-FOV HSRL detecting multiple scattering signals from seawater constituents.

The triple-FOV HSRL system, deployed on the foredeck of R/V Runjiang No.1, is composed of the upper and lower parts (Fig. 1A). The transmitter and receiver are integrated into the upper part to ensure a stable optical system with supporting devices in the lower part. The transmitter, emitting the laser pulse into the seawater, mainly consists of a diode-pumped, Q-switched, injection-seeded, frequency-doubled neodymium-doped yttrium aluminum garnet laser with a pulse energy of 10 mJ at 532 nm and a repetition frequency of 10 Hz (Fig. 1B) [19–21]. The laser wavelength is locked to the absorption line of an iodine cell by a proportional-integral-derivative (PID) servo loop. The lidar signals, including particulate scattering and molecular Brillouin scattering, are collected through a telescope and divided into 4 channels. The combined channel at the wide FOV (200 mrad) detects all components of the lidar signals, while the 3 molecular channels at the triple-FOV (40, 80, and 200 mrad) all reject the particulate signal and transmit the molecular Brillouin signal through the exploitation of the iodine absorption line. Photomultiplier tubes (PMT) and a high-speed data acquisition card are used to record the lidar signals [22,23]. The channel gains are intercalibrated by the gain ratio calibration of the PMTs. The main parameters of oceanic triple-FOV HSRL are shown in Supplementary Materials (Table S3), and the optimization of lidar FOVs is analyzed in Text S1.

The basic principle of triple-FOV HSRL for detecting seawater constituents depends on the multiple scattering of photons in the seawater (Fig. 1C). The emitted photons of the laser beam can be scattered in different directions by seawater constituents. In this process, multiple scattering takes place when a portion of scattered photons is dispersed again by neighbouring molecules or particles. Hence, lidar signals are generated by combining single scattered and multiple scattered photons collected by the telescope. In particular, the expansion of the signal angular distribution due to multiple scattering results in intensity variations collected by different FOVs. These signals are governed by the absorption coefficient, the scattering coefficient, the PSD of particles, and the spatial geometry distribution of the observation using the analytical model (see the Materials and Methods for details). Therefore, significant information of seawater constituents can be obtained from the multi-FOV system.

**Fig. 2.**
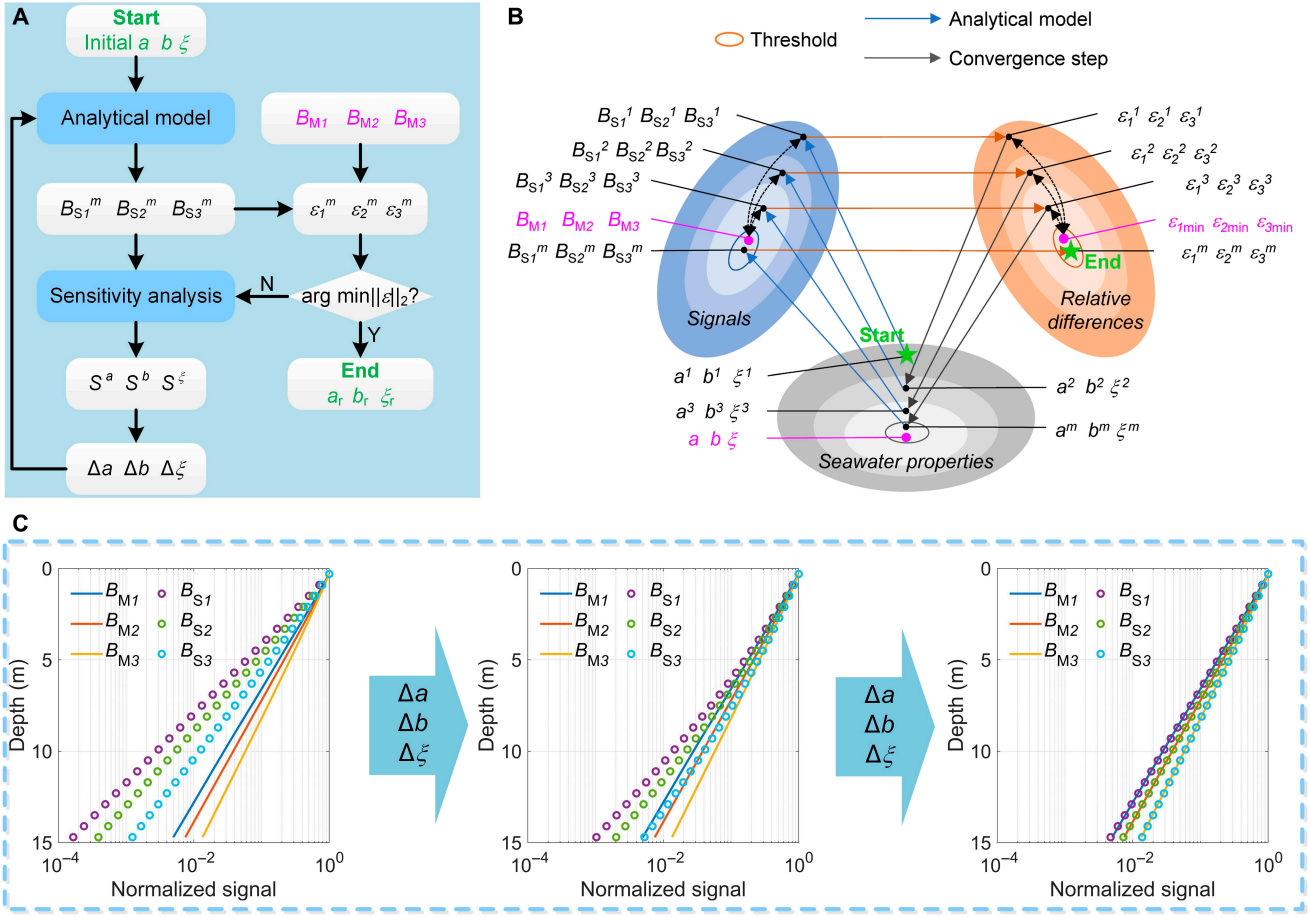
Triple-FOV HSRL retrieval algorithm. (A) Flow chart of the retrieval algorithm. (B) Schematic diagram of the iteration process. (C) Example of the signal iteration process. *ε* is the relative difference between the simulated and measured molecular signals *B*_S_ and *B*_M_. The superscripts 1, 2, 3...*m* is the number of iterations.

The seawater absorption coefficient (*a*), the scattering coefficient (*b*), and the slope of PSD (*ξ*) (see the Materials and Methods for more details) are retrieved from triple-FOV molecular signals according to an iterative retrieval scheme (Fig. 2A). The steps are as follows:

1. The simulated molecular signals (*B*_S_) are produced by using the analytical model as mentioned above. The initial values of *a*, *b*, and *ξ* input into the model are estimated as the lidar attenuation coefficient at wide FOV, narrow FOV, and a median value within the possible range of coastal water [24], respectively.

2. An iterative retrieval scheme is employed to retrieve the most likely values of *a*, *b*, and *ξ* by minimizing the relative differences between the simulated signal *B*_S_ and the measured molecular signal *B*_M_ at triple FOVs. This process can be expressed as a numerical solution to the minimum of the 2-Norm form, that is,

$$\begin{array}{c} X=\left[a_{r},b_{r},\xi_{r}\right]=\text{arg}\text{min}{\left\|\frac{\Delta B}{B}\right\|}_{2},\\ \frac{\Delta B}{B}=\left[\frac{B_{M1}-B_{S1}}{B_{M1}},\frac{B_{M2}-B_{S2}}{B_{M2}},\frac{B_{M3}-B_{S3}}{B_{M3}}\right], \end{array}$$ (1)

where *a*_r_, *b*_r_, and *ξ*_r_ are the retrieved values of *a*, *b*, and *ξ*, respectively; the subscripts *1*, *2*, and *3* correspond to the narrow, medium, and wide FOV, respectively. The signal relative differences are gradually approaching the minimum value, while *a*, *b*, and *ξ* are adjusted toward true values during the convergence iterations (Fig. 2B). Taking a trial group at the *j*th iteration of *a_j_*, *b_j_*, and *ξ_j_* as an example, the retrieval parameters are perturbed as **X**_*j*+1_ =**X***_j_* + Δ**X***_j_*. The convergence steps Δ**X***_j_* are generated by:

$$\frac{\Delta X}{X}=\frac{\Delta B}{B}S^{-1},$$ (2)

where **S** is the triple-FOV signal sensitivity to *a*, *b*, and *ξ* (see the Supplementary Materials, Text S1 for more details), calculated by the analytical model. Due to signal noises, the iteration is forced to stop when the relative difference is below a threshold value predetermined by the system noise levels. In this work, 10% root-mean-square relative difference between the simulated *B*_S_ profile with the measured *B*_M_ profile is used, and its detailed definition is given in the next section. It should be noted that the seawater characteristics at different depths corresponding to the specific profiles under 3 FOVs are retrieved simultaneously. It is because that the lidar signal at a deeper depth is jointly determined by the seawater characteristics at all depths above it.

To illustrate the iteration process, an intuitive example is given (Fig. 2C): the initial values of *B*_M_ and *B*_S_ corresponding to the initial values of *a*, *b*, and *ξ* at different FOVs are provided (left plot); combining the signal differences and sensitivities in Eqs. 1 and 2, values of ∆*a*, ∆*b*, and ∆*ξ* resulting in convergence of *B*_S_ to *B*_M_ are derived (middle plot); finally, the relative differences fall below the threshold values, and the retrieval results are obtained (right plot).

In addition to *a*, *b*, and *ξ*, the particulate backscatter coefficient (*b*_bp_) and *K*_d_ can be obtained from the combined and molecular signals at the wide FOV [25]. Similar to the atmospheric aerosol studies [26–29], the lidar ratio (*R*) and the single scattering albedo (*ω*_0_) are regarded as key parameters for the characterization and classification of seawater constituents. *R* is defined as the ratio of *K*_d_ excluding pure seawater to the particulate volume scattering function at 180°, which can be obtained by a single-FOV HSRL. *ω*_0_ is the ratio of the particulate scattering coefficient to the beam attenuation coefficient excluding pure seawater, which can be calculated from the triple-FOV HSRL retrieval products.

### Performance of the triple-FOV HSRL technique

**Fig. 3.**
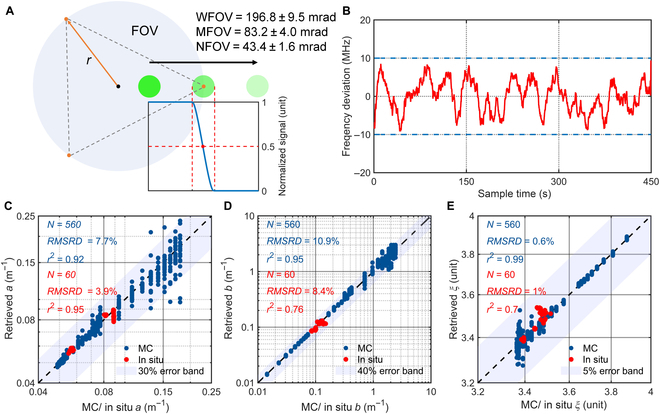
The performance of the triple-FOV HSRL technique. (A) Calibration of FOVs. (B) Locked laser frequency drift compared with the iodine line. Comparisons of (C) the absorption coefficient *a*, (D) the scattering coefficient *b*, and (E) the slope of PSD *ξ* with MC and in situ results. WFOV, MFOV, and NFOV are for the wide FOV, middle FOV, and narrow FOV, respectively.

To ensure the quality of measurements, the edge and width of the triple FOVs are calibrated by the laser spot position where the lidar signal attenuates to half (see the Supplementary Materials, Text S2 and Fig. S1). As shown in Fig. 3A, the final calibration values for the triple-FOV are 196.8 ± 9.5 mrad (wide FOV), 83.2 ± 4.0 mrad (middle FOV), and 43.4 ± 1.6 mrad (narrow FOV), respectively, proving the accuracy of the system FOV design. By the PID servo loop, the laser frequency is locked to the iodine absorption line within an offset frequency of 10 MHz (Fig. 3B). In this way, coincident wavelengths of the laser emission, the iodine line, and the particulate backscatter signals are generated to ensure the retrieval accuracy of optical properties at 532 nm.

Moreover, simulated data from the MC technique and in situ data from field measurements are used to verify the consistency of the triple-FOV HSRL results. Parameter settings of the MC simulations (see the Supplementary Materials, Tables S1 and S2) and profiles of field experiments (see the Supplementary Materials, Fig. S2) are given for more details. To evaluate the relative difference between *B*_S_ with *B*_M_ or the retrieval accuracy, the root-mean-square relative difference (*RMSRD*) is used, which is defined as

$$\text{RMSRD}=\sqrt{\frac{\sum_{i=1}^{n}{\left(x_{i}/{\tilde{x}}_{i}-1\right)}^{2}}{n}}\times 100\%,$$ (3)

where *n* is the total number of signal sampling bins; *x_i_* is the simulated or lidar retrieved values; ${\tilde{x}}_{i}$ is the MC input or measured values, working as the reference values.

The lidar measurements of *a*, *b*, and *ξ* are found to be in good agreement with MC and in situ estimates (Fig. 3C to E). Estimated values of *RMSRD* for *a*, *b*, and *ξ* between the triple-FOV HSRL and MC values are 7.7%, 10.9%, and 0.6%, respectively. A strong correlation is observed with a coefficient of determination (*r*^2^) of 0.92, 0.95, and 0.99, respectively. Relative differences are higher in turbid seawaters, i.e., when *a* and *b* are larger (*ξ* is smaller), since the signal fluctuations of the MC simulations are more significant in this case. In field experiments, values of *RMSRD* are 3.9%, 8.4%, and 1.0%, with values of *r*^2^ as 0.95, 0.76, and 0.70, respectively, for *a*, *b*, and *ξ* constituting an excellent agreement when compared with in situ measurements.

### Underway measurements

**Fig. 4.**
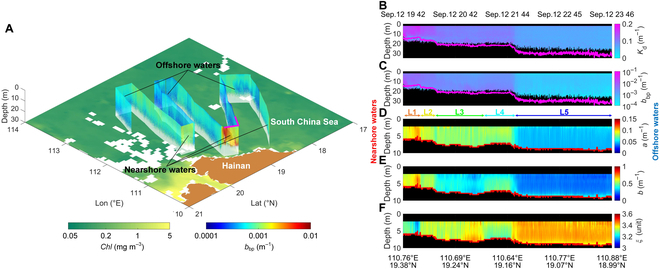
Triple-FOV HSRL and ocean color results near Hainan Province on 2020 September 12. (A) Continuous S-shaped underway triple-FOV HSRL measurements of the particulate backscattering coefficient *b*_bp_ together with the surrounding chlorophyll-a concentration *Chl* from MODIS satellite measurements. The magenta line shows the area selected for further studies. (B) Diffuse attenuation coefficient *K*_d_. (C) Particulate backscattering coefficient *b*_bp_. (D) Absorption coefficient *a*. (E) Scattering coefficient *b*. (F) Slope of PSD *ξ*. Magenta lines in (B) and (C) indicate 3 optical depths, and red lines in (D) and (E) show the maximum effective depth of the triple-FOV HSRL retrieval, which is mainly determined by the noise level of the signal at the narrow FOV, where the dynamic range of 3 orders of magnitude is selected in this work.

During the 2020 Joint autumn cruise, underway measurements were carried out from September 11 to September 13 (UTC+0800) with the ship track and study region in the South China Sea (see the Supplementary Materials, Fig. S3). Profiles of *b*_bp_ from the triple-FOV HSRL are plotted in Fig. 4A. The results show a decreased trend of turbidity from nearshore to offshore, which is consistent with variations of chlorophyll-a concentration (*Chl*) from Moderate-resolution Imaging Spectroradiometer (MODIS) monthly products. High values of *b*_bp_ and *Chl* in the nearshore water are attributed to the sufficient nutrient supply due to the shallow depth and prevalent upwelling near Hainan Island [30]. The transect with distinct nearshore-to-offshore gradients of *b*_bp_ on September 12 was selected for further investigation (magenta line in Fig. 4A).

Along the transect, depth-resolved distributions of *K*_d_, *b*_bp_, *a*, *b*, and *ξ* were obtained except near the sea surface (0 to 2 m, to avoid surface effect) and at depths where signal-to-noise ratios are too low (Fig. 4B to F). The transect is divided into 5 regions, L1 to L5, based on the variation of seawater characteristics. In the L1 region that is closest to the shore, *K*_d_, *b*_bp_, *a*, and *b* reached maximum values of about 0.17, 0.005, 0.11, and 0.75 m^−1^, respectively, while *ξ* came to the minimum value of about 3.15. As the ship traveled southwest into the L2 region, *K*_d_, *b*_bp_, *a*, and *b* decreased to 0.12, 0.0028, 0.085, and 0.55 m^−1^, respectively, while *ξ* increased to 3.35. Moving offshore, *K*_d_, *b*_bp_, *a*, and *b* decreased rapidly to about 0.1, 0.0018, 0.078, and 0.4 m^−1^, respectively, in the junction area of L2 and L3, while *ξ* remained relatively constant. In the L3 region, seawater was clearer than in the L1 to L2 region, with *b*_bp_ and *b* gradually decreasing with increasing *ξ*. Then the ship turned the direction near the L4 region, during which the ship first sailed to nearshore and then headed offshore to the southeast. This trajectory was reflected in the fact that *b*_bp_, *b*, and *ξ* came to about 0.002 m^−1^, 0.45 m^−1^, and 3.3 in the L4 region, then *K*_d_, *b*_bp_, *a*, and *b* decreased to their minimum values in the L5 region (about 0.07, 0.00045, 0.047, and 0.25 m^−1^, respectively), while *ξ* achieved a maximum value of about 3.42. In addition, vertical variations of characteristics were found near the water surface in the L4 regions, indicating the higher concentration of seawater constituents at greater depths. These results demonstrate that the water clarity gradually increases from the nearshore to the offshore region, which is consistent with other studies [31,32]. *ξ* was negatively correlated with the other seawater optical properties (see the Supplementary Materials, Fig. S4), indicating that the slope of the PSD decreases as the proportion of large particles increases in more turbid seawater [33]. This series of long-distance observations showcase the utility of the triple-FOV HSRL technique providing a continuous observation of seawater characteristics.

### Constituent classification and carbon export

**Fig. 5.**
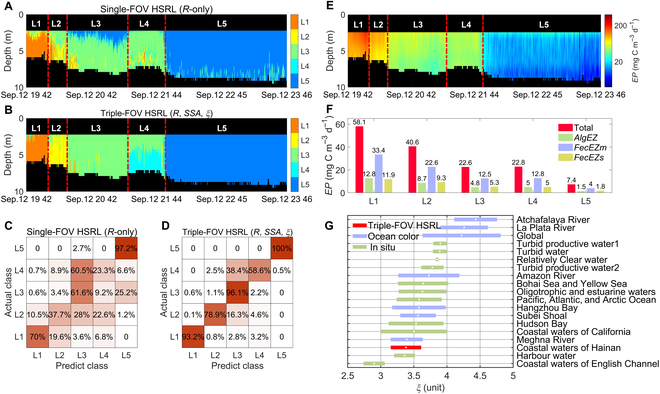
Applications of retrieval results. (A) Classification results from single-FOV HSRL. (B) Classification results from triple-FOV HSRL. (C) Confusion matrix of single-FOV HSRL classification. (D) Confusion matrix of triple-FOV HSRL classification. (E) Depth-resolved particulate organic carbon export flux *EP*. (F) Contributions of different sources to *EP* in L1 to L5 regions; *AlgEZ* is the direct sinking export of large phytoplankton; *FecEZm* is the flux of fecal matter from large zooplankton grazing; *FecEZs* is the flux of fecal matter from small zooplankton grazing. (G) Historical *ξ* values from previous studies, including this work; the white center point of the color bar represents the mean value, and the lengths on both sides represent the SD of the observations.

As demarcations of different regions are clearly visible, it is fairly appealing to employ optical properties and PSD for seawater constituent classification. The lidar ratio (*R*), the single scattering albedo (*ω*_0_), and *ξ* are clustered (see the Supplementary Materials, Fig. S5) and classified (Fig. 5A to D) for L1 to L5 regions using the random-forest-based seawater classification model (see the Materials and Methods for more details). It exhibits a substantial improvement compared to using only *R* based on the single-FOV HSRL for classification (Fig. 5A and B). In particular, single-FOV HSRL classification results in a large confused proportion in the L2-L4 regions between nearshore and offshore. Notably, the triple-FOV HSRL method improved the self-validation accuracy of classification confusion matrix in these 3 regions by 2.1, 1.6, and 2.5 times, respectively (Fig. 5C and D). The triple-FOV HSRL classification is able to resolve a shallow L3 in the L4 region, presenting the intrusion of seawater from the L3 region into the surface layer of the L4 region (Fig. 5B). A possible explanation for this intrusion event might be that the tide impacts the diurnal variation in seawater constituents [34]. The night-time high tide near Hainan Island (see the Supplementary Materials, Fig. S6) carried deeper seawaters of the L3 region into the upper layer of nearshore seawaters, causing marked stratifications of seawater characteristics in the L4 region. The results indicate that additional characteristics benefit the classification of seawater constituents and the discovery of special phenomena.

Particles of biological origin play an important role in ocean carbon sequestration by converting inorganic carbon into organic carbon and exporting particulate organic carbon to the deep ocean [35]. However, estimates of the particulate organic carbon export flux (*EP*) remain highly uncertain, mainly limited to surface-integrated quantities [36]. The PSD slope *ξ* provides ways to compute the particle size fractions relative to total particles [24]. A depth-resolved *EP* can be computed by employing the *K*_d_, *b*_bp_, and *ξ* profiles with other auxiliary data based on the food-web model (see the Materials and Method).

Overall, *EP* showed similar distribution patterns as those of seawater characteristics along the transect from the nearshore to offshore regions (Fig. 5E). The intensity of *EP* decreased from a maximum of 58.08 mgCm^−3^d^−1^ in the L1 region to a minimum of 7.37 mgCm^−3^d^−1^ in the L5 region (Fig. 5F). The results suggest that values of *EP* were dominated by the flux of fecal matter from zooplankton grazing (*FecEZ*) in all regions, of which the large phytoplankton (*FecEZm*) took a more significant fraction than the small phytoplankton (*FecEZs*), while the direct sinking export of large phytoplankton (*AlgEZ*) made a much smaller contribution [36]. High values of *EP* were found in parts of the sea surface in the L1 region, perhaps due to the aggregation of nearshore phytoplankton and other organic particles. This result was consistent with the *Chl* and seawater characteristics distributions (Fig. 4). Besides, phytoplankton will accumulate in shallow waters because of stronger light intensity, which may lead to the decrease of *NPP* and *EP* with depth increasing in nearshore seawaters [37]. As mentioned previously, the high tide from L3 took part of phytoplankton away from the upper L4, and reduced the *EP* of surface seawaters and weaken the decreasing trend of *EP* with depth in the L4 region. This intrusion event was also reflected in the more significant contributions of large phytoplankton (*AlgEZ* and *FecEZm*) into the *EP* than in the surrounding regions. The depth-resolved *EP* derived from triple-FOV HSRL measurements offers vertical information on carbon exports and will further advance studies on the 3-dimensional global carbon cycle.

Numerous studies have attempted to obtain PSD slope based on remote sensing of ocean color or directly from in situ measurements. Previous values of *ξ* range from 2.5 to 5 [31–33,38–46], with typically smaller values in turbid waters compared to higher values in clear seawaters (Fig. 5G). The mean value and SD of *ξ* from triple-FOV HSRL are found to be 3.38 and 0.23 in this work, respectively. The values are significantly smaller than values in clear open ocean environments (around 4.0). We have found that the relatively low values of *ξ* in the coastal seawater near Hainan are consistent with the characteristics of the more turbid waters in other studies, such as the coastal waters of California [31] or the English Channel [40], potentially indicating a more significant *EP* than other clearer regions.

## Discussion

In this work, we developed a novel oceanic triple-FOV HSRL that can provide comprehensive, continuous, and vertical measurements of seawater constituents. This high-performance technique can retrieve multiproperty profile measurements (absorption coefficient *a*, scattering coefficient *b*, and slope of PSD *ξ*) in various water types, and we have validated these abilities using both MC radiative transfer simulations and in situ measurements with high fidelity (Fig. 3). Furthermore, we studied a typical nearshore-to-offshore transect in the coastal seawater of Hainan Island on 2020 September 12 (Fig. 4). Although ocean color remote sensing data, such as MODIS and Visible infrared Imaging Radiometer products, contain global surface-integrated optical properties and particle size parameters of seawaters [9,33], the triple-FOV HSRL provides more refined depth-resolved characteristics of seawater constituents for conducting in-depth analyses of shallow oceans. The continuous profiles obtained by the triple-FOV HSRL reveal both horizontal gradients and vertical variations of comprehensive seawater constituent characteristics, which can be very difficult to get from in situ measurements, such as the WETLabs AC-S and HOBILabs HS6P used in this work.

Using the triple-FOV HSRL technique, we developed a seawater classification approach based on lidar ratio (*R*), single scattering albedo (*ω*_0_), and *ξ* (Fig. 5A to D). The accuracy of self-validation in the L1 to L5 region is improved by up to 41.2% compared to the results for *R*-only classification. An interesting seawater layer intrusion event related to the tide impact on the variation of seawater constituents was founded in the classification results. In addition, triple-FOV HSRL can provide additional seawater characteristics for other classification schemes, such as remote-sensing-reflectance-based [47] and *K*_d_-based [48] methods, producing a more accurate and informative seawater classification. In this work, we have not distinguished between planktonic and mineral fractions of oceanic particles. The integration of polarization information [49] available with triple-FOV HSRL will enable us to further classify particulate constituents of upper seawaters. Distinguishing the contribution of different plankton or particle types is imperative for validating and improving the food-web model processes and ocean carbon cycle analyses.

In the global carbon cycle, the biological carbon pump by seawater constituents is essential for ocean carbon sequestration by converting inorganic source into organic carbon and exporting it to the deep ocean, and it is an important scientific quest to study the link between this process to the changing climate [2]. However, there remains great challenges to getting accurate estimates of *EP* and its vertical distribution based on ocean color remote sensing or in situ measurements [50]. The *ξ* from the triple-FOV HSRL can be used to constrain the microphytoplankton contribution to the total chlorophyll-a concentration, which is a critical parameter in the estimation of *EP*. In combination with auxiliary data obtained from other instruments, the triple-FOV HSRL enables the analysis of depth-resolved *EP* and its components in different regions, indicating the great potential of this technique in improving ocean carbon studies (Fig. 5E and F).

Promising future directions also include improving the system detection capabilities, accumulating more field experiment data, and integrating the triple-FOV HSRL with other measurement systems to benefit future investigations into plankton community structure and succession. Overall, the proposed triple-FOV HSRL technique can provide an improved solution for interpreting seawater constituent characteristics and expanding our understanding of marine ecosystems and biogeochemistry.

## Materials and Methods

### Parameterization of the seawater

The absorption coefficient (*a*), scattering coefficient (*b*), and backscattering coefficient (*b*_b_) can be separated into the contribution from pure seawater and other components, where the values for pure seawater at 532 nm adopted in this work are 0.042, 0.0022, and 0.0011 m^−1^, respectively [51,52]. The effects of temperature and salinity were omitted. Optical properties are determined by the particulate constituents and their PSD in seawaters.

Several analytical models for the PSD of seawater particles have been proposed in previous studies. The most used PSD is a power-law type [53]:

$$Np\left(D\right)=N_{0}{\left(\frac{D_{0}}{D}\right)}^{-\xi },$$ (4)

where *N*_p_ is the number concentration of particles per volume of seawater particles in m^−3^μm^−1^; *D* is the particle diameter in μm; *N*_0_ is the particle number concentration at the reference diameter *D*_0_, and *ξ* is the power-law exponent of the PSD (often referred to as “slope”). As described by this power-law distribution, *N*_p_ decreases with the increase of *D*, consistently with the analysis of the ecological structure and predator–prey relationships in planktonic ecosystems [54]. The distribution and evolution of the PSD are closely related to physical and biogeochemical processes in seawaters. The Fourier–Forand phase function [53] provides us with an analytic expression for the phase function of a population of homogeneous spheres, distributed according to the power-law size distribution based on a modified form of the anomalous diffraction approximation. This function is used in our calculations of the further multiple scattering model and iterative retrieval process. Basing on the Fourier–Forand phase function, the particulate backscattering fraction of the seawater *BF* can be further obtained by

$$\mathrm{BF}=\frac{b_{\mathrm{bp}}}{b_{p}}=1-\frac{1-\delta_{90}^{v+1}-0.5\left(1-\delta_{90}^{v}\right)}{\left(1-\delta_{90}^{v}\right)\delta_{90}^{v}},$$ (5)

where *b*_p_ is the particulate scattering coefficient in m^−1^; *δ*_90_ = 0.75(*m* − 1)^−2^sin^2^(90°/2) and *v* = (3 − *ξ*)/2 are intermediate variables; *m* = 1.01 + 0.1542(*ξ* − 3) represents the refractive index of particles relative to seawater.

### Multiple scattering model

The analytical model based on the quasi-single-scattering small-angle approximation is used to simulate oceanic HSRL signals efficiently [55]. The analytical model is applied to the forward simulation of triple-FOV HSRL signals and the further iterative retrieval process, achieving more credible simplification than the traditional methods. In the analytical model, the coaxial molecular signal profile of HSRL can be expressed as

$$B_{\mathrm{Sn}}=F\left\{C_{0},\left[a_{1}\ldots a_{i}\right],\left[b_{1}\ldots b_{i}\right],\left[\xi_{1}\ldots \xi_{i}\right],{\mathrm{FOV}}_{n}\right\},$$ (6)

where *F* represents the forward simulation of HSRL molecular signals by analytical model; *C*_0_ is the system constant; *a*_1_…*a_i_*, *b*_1_…*b_i_*, and *ξ*_1_…*ξ_i_* are whole profiles of *a*, *b*, and *ξ*. This means that once the lidar system parameters are determined, the molecular signal can be written as an equation of *a*, *b*, and *ξ*.

Data from a semianalytical MC were used to validate the performance of the retrieval algorithm in this work. The details of this model can be found in our previous work [56]. The traditional MC applied to lidar achieves the purpose of signal simulations by tracing the scattering process of numerous photons in the water and counting the number of photons returned to the lidar receiver. The semianalytical MC model combines the statistical approach and the analytical estimation, which greatly improves the efficiency of the MC simulation and reduces the fluctuation of the simulated signal. To obtain the multichannel signals in triple-FOV HSRL simultaneously, the semianalytical MC considered in this paper relies on both particulate scattering and molecular scattering and divides the lidar returns into combined channels and molecular ones.

### In situ measurements

An optical package, which included WETLabs AC-S and HOBILabs HS6P, measuring in situ optical properties was deployed at specific locations (stations) lowered with a winch. WETLabs AC-S can measure the spectral absorption and beam attenuation coefficients (here, we use only the values at 532 nm). The particulate backscattering coefficient at 532 nm was obtained from the particulate backscattering at 510 nm from HOBILabs HS6P, assuming *b*_bp_(*λ*) = *b*_bp_(*λ*_0_)*λ*_0_/*λ* [57]. *ξ* is calculated by substituting the in situ values of *b*_p_ and *b*_bp_ into Eq. 5. All in situ data were binned to a depth resolution of 1 m.

It should be noted that, in situ *ξ* are converted from in situ measurements of the particulate backscattering fraction *BF* of the seawater. Therefore, for the rigor, we also converted retrieved *ξ* to *BF* and compared them with in situ values in the Supplementary Materials, Fig. S2.

### Assessment of particulate organic carbon export flux

Vertically resolved particulate organic carbon export flux (*EP*) is defined using a 2-component food-web-based model as [36,50]

$$\mathrm{EP}\left(z\right)=\text{AlgEZ}\left(z\right)+\text{FecEZ}\left(z\right),$$ (7)

where *AlgEZ* is the direct sinking export of large phytoplankton and *FecEZm* is flux of fecal matter from zooplankton grazing. *AlgEZ* can be calculated as the product of the net primary production (*NPP*). This model has been commonly used in the studies of *EP* [36,50], and the triple-FOV HSRL data are applied to it in this work. The *NPP* is calculated using the carbon-based production model, where inputs include *b*_bp_ (from triple-FOV HSRL), chlorophyll-a (*Chl*, derived by *K*_d_ from triple-FOV HSRL), and photosynthetically available radiation (*PAR*, from MODIS L3 monthly products) [58].

The *FecEZ*, which can be divided into parts from large (*FecEZm*) and small phytoplankton (*FecEZs*), is calculated using the *NPP*, *b*_bp_, *K*_d_, the rate of change in *Chl* (from MODIS L3 products) and mixed-layer depth (calculated from wind field data of CMPP 2.0) [50,59]. The fractions of micro and small phytoplankton are estimated based on the value of *ξ* [24].

### Seawater classification method

Random forest is one of the most successful methods currently applied to various regressions, classifications, and other related issues [60]. A random forest classification model is used for the seawater classification in this work, in which a total of 100 trees with a split number of 5 and a depth of 12 are adopted. *R*, *ω*_0_, *ξ* (or only *R*), and corresponding seawater types L1 to L5 work for the model training. Finally, confusion matrixes of self-validation are used to evaluate the classification performance.

## Data Availability

The digital topographic data from the ETOPO1 Global Relief Model published by the National Geophysical Data Center are available at http://www.ngdc.noaa.gov/mgg/global/global.html. The tide height data from the National Marine Data and Information Service are available at http://global-tide.nmdis.org.cn. The Chl and PAR from Level-3 products of MODIS are available at https://oceancolor.gsfc.nasa.gov/l3. The wind field data from CCMP Wind Vector Analysis Product (L3, version 2.0) are available at https://data.remss.com/ccmp. The code of the random forest method is available at https://github.com/karpathy/Random-Forest-Matlab. The other data are available from the corresponding author upon reasonable request.
