# Supplementary material for: Comprehensive, Continuous, and Vertical Measurements of Seawater Constituents with Triple-Field-of-View High-Spectral-Resolution Lidar
Source: Research (Wash D C). 2023 Jul 19;6:0201. doi: 10.34133/research.0201 (PMC10355187; doi:10.34133/research.0201)
Supplement: Supplementary 1 — Text S1 and S2 Figs. S1 to S8 Tables S1 to S4 [file research.0201.f1.docx]

Supplementary materials for

**Comprehensive, continuous, and vertical measurements of seawater constituents with triple-field-of-view high-spectral-resolution lidar**

Kai Zhang^1,2^†, Yatong Chen^1,3^†, Hongkai Zhao^1^, Zhongping Lee^4^, Emmanuel Boss^5^, Iwona Stachlewska^6^, Davide Dionisi^7^, Cédric Jamet^8^, Paolo D. Girolamo^9^, Aleksey Malinka^10^, Chengchong Jiang^1^, Hongda Wu^1^, Lingyun Wu^1^, Feitong Chen^1^, Xiaolei Zhu^11^, Nanchao Wang^1^, Chuxiao Chen^1^, Qun Liu^1^, Lan Wu^1^, Yudi Zhou^1^*, Weibiao Chen^11^*, and Dong Liu^1, 2, 12, 13^*

1 Ningbo Research Institute, State Key Laboratory of Modern Optical Instrumentation, College of Optical Science and Engineering, Zhejiang University, Hangzhou 310027, China

2 ZJU-Hangzhou Global Scientific and Technological Innovation Center, Zhejiang University, Hangzhou 311200, China

3 Donghai Laboratory, Zhoushan, 316021, China

4 State Key Lab of Marine Environmental Science, College of Ocean and Earth Sciences, Xiamen University, Xiamen 361102, China

5 School of Marine Sciences, University of Maine, ME 04469-5741, USA

6 Faculty of Physics, University of Warsaw, Warsaw 02093, Poland

7 Institute of Marine Sciences, Italian National Research Council, Rome 00133, Italy

8 Université du Littoral Côte d'Opale, CNRS, Univ. Lille, IRD, UMR 8187 - LOG - Laboratoire d'Océanologie et de Géosciences, Wimereux F-62930, France

9 Institute Scuola di Ingegneria, Università della Basilicata, Potenza 85100, Italy

10 Institute of Physics, National Academy of Sciences of Belarus, Minsk 220072, Belarus

11 Key Laboratory of Space Laser Communication and Detection Technology, Shanghai Institute of Optics and Fine Mechanics, Chinese Academy of Sciences, Shanghai 201800, China

12 Intelligent Optics & Photonics Research Center, Jiaxing Research Institute Zhejiang University, Jiaxing 314000, China

13 Jiaxing Key Laboratory of Photonic Sensing & Intelligent Imaging, Jiaxing 314000, China.

*Address correspondence to: Yudi Zhou; zhouyudi@zju.edu.cn, Weibiao Chen; wbchen@siom.ac.cn, and Dong Liu; liudongopt@zju.edu.cn.

† These authors contributed equally to this work.

This document includes Supplementary Text S1–S2, Supplementary Fig. S1–S8, and Supplementary Table S1–S4.

# **Supplementary** **Texts**

**Supplementary Text S1. The optimization of lidar FOVs**

As a key factor of the triple-FOV HSRL technique, the widths of FOVs need to be optimized for the system design. The optimization of the triple-FOV aims to achieve high sensitivities of molecular channel signals to *a*, *b*, and *ξ*, respectively, which ensures the stability and accuracy of retrieval products. In this study, signal sensitivities and FOV optimizations were analyzed with parameter settings of our shipboard triple-FOV HSRL (same as Table S3 except for FOVs). The optimization of FOVs for other similar systems or detection scenarios can be performed with the same strategy, which will not be repeated here. The triple-FOV signal sensitivities to *a*, *b*, and *ξ* can be defined as

$$\mathbf{S}=\left[\begin{matrix} \frac{a}{B_{\text{S}1}}\frac{\partial B_{\text{S}1}}{\partial a} & \frac{a}{B_{\text{S}2}}\frac{\partial B_{\text{S}2}}{\partial a} & \frac{a}{B_{\text{S}3}}\frac{\partial B_{\text{S}3}}{\partial a} \\ \frac{b}{B_{\text{S}1}}\frac{\partial B_{\text{S}1}}{\partial b} & \frac{b}{B_{\text{S}2}}\frac{\partial B_{\text{S}2}}{\partial b} & \frac{b}{B_{\text{S}3}}\frac{\partial B_{\text{S}3}}{\partial b} \\ \frac{\xi}{B_{\text{S}1}}\frac{\partial B_{\text{S}1}}{\partial\xi} & \frac{\xi}{B_{\text{S}2}}\frac{\partial B_{\text{S}2}}{\partial\xi} & \frac{\xi}{B_{\text{S}3}}\frac{\partial B_{\text{S}3}}{\partial\xi} \end{matrix}\right],$$

In Fig. S7, the sensitivities of molecular signals at 10 meter depth to *a*, *b*, and *ξ* are shown. As presented in Fig. S7A, the signal sensitivities to *a* keep constant at different FOVs, i.e., any FOV has sufficient sensitivity to *a* theoretically. Therefore, we chose the wide FOV of 200 mrad to improve the signal-to-noise ratio and the detection depth of *K*_d_ and *b*_bp_ with adequate sensitivity to *a*. The peak signal sensitivity to *ξ* corresponds to narrower FOVs in more turbid seawaters (Fig. S7B), so we chose the narrow FOV of 40 mrad, which is the most sensitive to the typical values of *ξ* (4.0) in most global seawaters[1]. The optimization of the middle FOV is a compromise. The sensitivity of the signal to *b* decreases monotonically with increasing FOV, as depicted in Fig. S7C. However, to facilitate subsequent retrievals, we chose 80 mrad as the middle FOV to significantly distinguish the signal from the other two FOVs, without losing much signal sensitivity. This choice, rather than a much narrower FOV, also ensures that the overlap factor (the geometrical overlap of the receiver FOV and the laser beam, varying from 0 to 1) reaches one above the water surface when the system is settled on the ship deck, avoiding the error introduced by the correction of the incomplete overlap signals at a narrower FOV.

**Supplementary Text S2. The calibration of lidar FOVs**

Different FOVs of the triple-FOV HSRL are constrained by the diaphragms on the focal plane of the lens, located very close to the PMTs. Despite the rigorous design of our system, the actual FOVs still deviate from the design values. Therefore, widths of triple-FOV need to be calibrated for the evaluation of FOV deviations. As shown in Fig. S1A, a convenient calibration method is adopted here:

(1) Adjust the laser emission direction and the optical axis of the FOV to be calibrated (FOV_c_) to the horizontal direction, and set a sizeable optical screen perpendicular to the optical axis at a distance *d* in front of the lidar system;

(2) Move the laser spot position on the optical screen into the FOV_c_ through adjustable reflectors and record the lidar signal amplitude *B*_max_;

(3) Move the spot to the edge of FOV_c_, and record the spot position *A_1_* when the lidar signal attenuates to 0.5*B*_max_;

(4) Move the spot back into FOV_c_, then adjust, and repeat step (3) in different spot moving directions to record two new edge points, *A_2_* and *A_3_*;

(5) Measure the distances among three points *A_1_*, *A_2_*, and *A_3_* as *l_1_*, *l_2_*, and *l_3_*, to determine the radius *r* of the projection of FOV_c_ on the optical screen. The width of FOV_c_ can be estimated as

where

$$r=\frac{l_{1}l_{2}l_{3}}{4\sqrt{p\left(p-l_{1}\right)\left(p-l_{2}\right)\left(p-l_{3}\right)}}$$

$$p=\left(l_{1}+l_{2}+l_{3}\right)/2$$

This calibration strategy has been used for all three FOVs, and the calibration was repeated three times for each FOV to minimize errors. The calibration results for the edge points of the triple-FOV HSRL are shown in the Fig. S1B and the measured data is listed in Table S4.

# **Supplementary Figures**


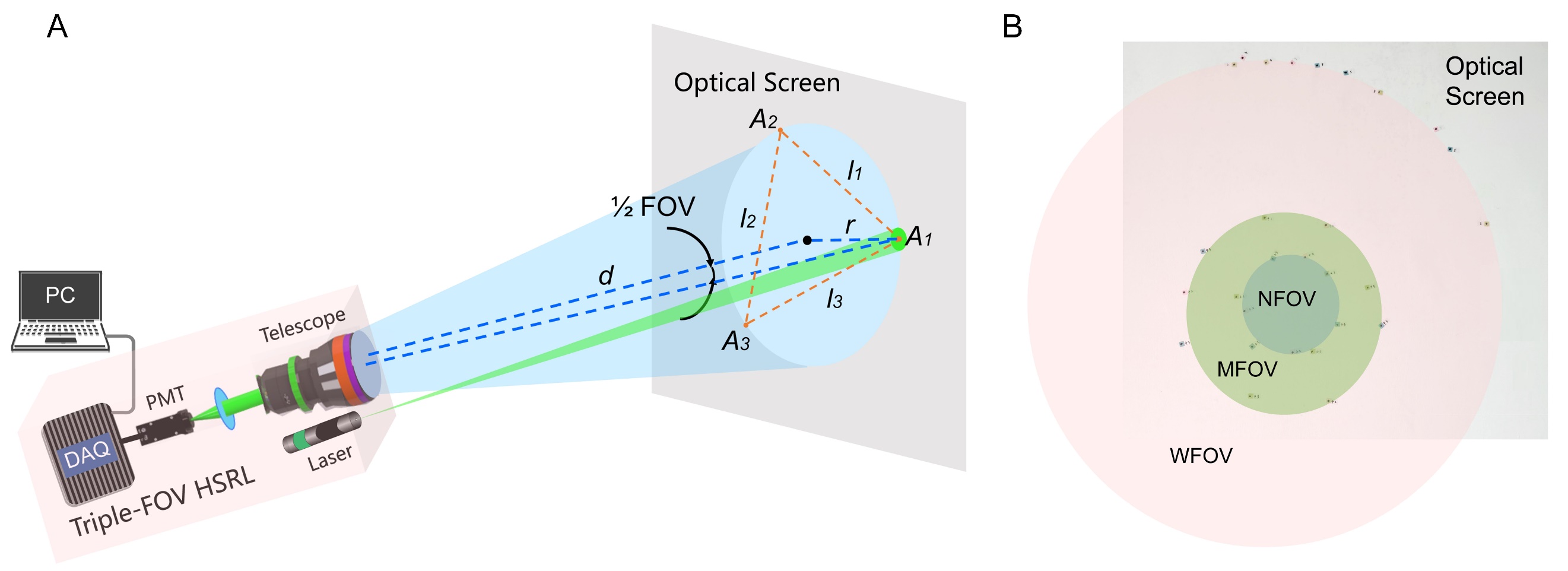


**Supplementary Fig. S1.** Calibration of FOVs. (A) Diagram of FOV calibration. (B) Calibrated edge points of the triple-FOV.


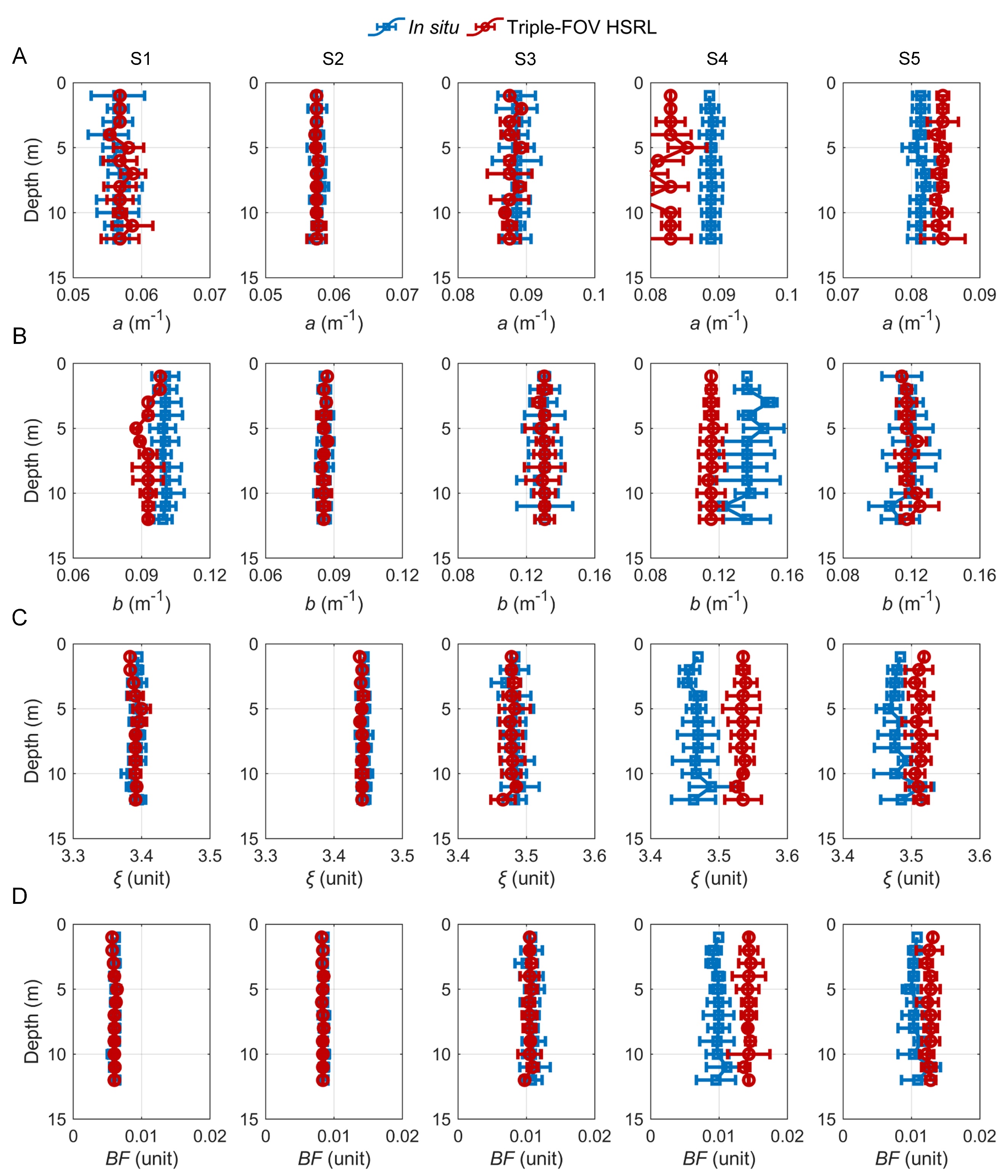


**Supplementary Fig. S2.** Profiles of seawater characteristics from the triple-FOV HSRL and in situ measurements at fixed stations S1-S5. (A) Absorption coefficient *a*. (B) Scattering coefficient *b*. (C) Slope of PSD *ξ*. (D) Particulate backscattering fraction of the seawater *BF*. S1 (20200907, 16:45, 117.32˚E, 22.50˚N); S2 (20200908, 10:47, 114.82˚E, 21.58˚N); S3 (20200914, 04:00, 110.65˚E, 18.75˚N); S4 (20200914, 06:00, 110.65˚E, 18.75˚N); S5 (20200914, 14:00, 110.65˚E, 18.75˚N).


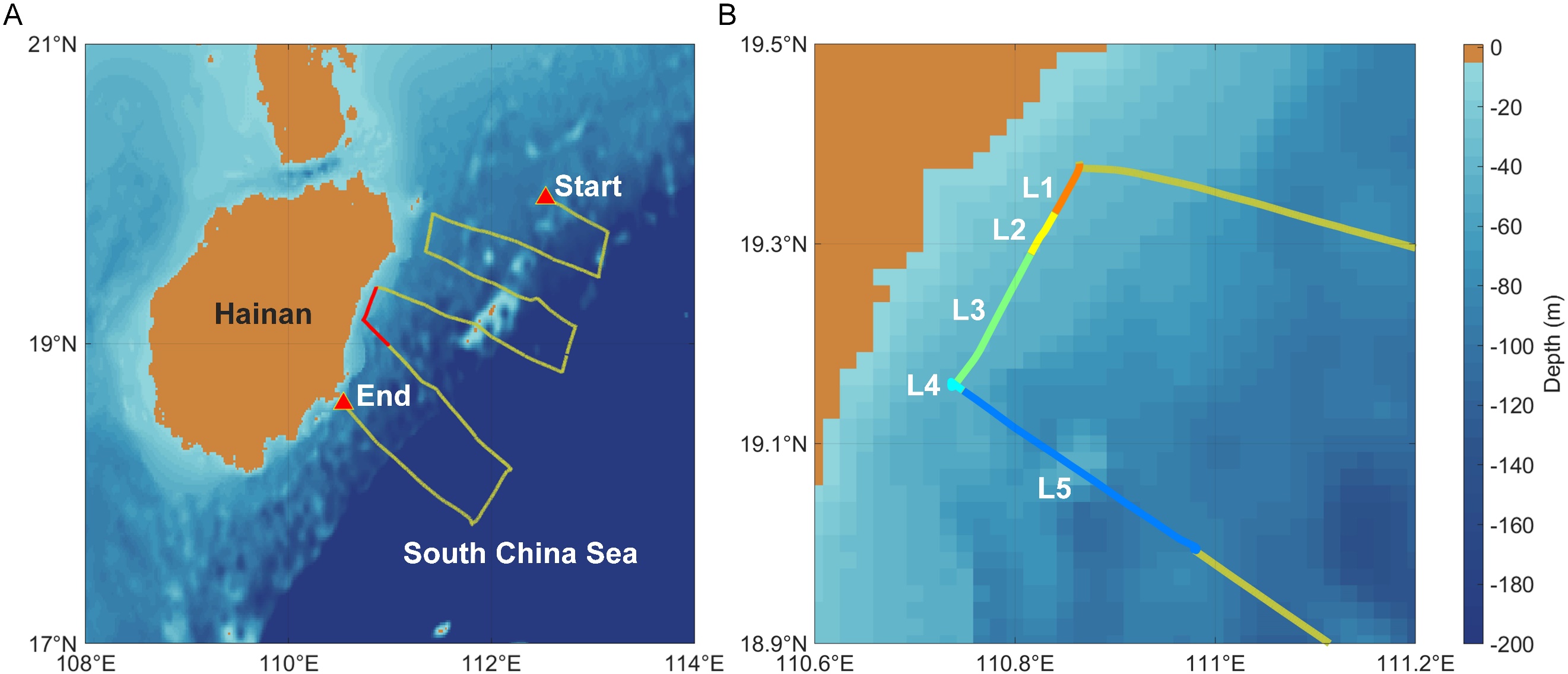


**Supplementary Fig. S3.** Continuous S-shaped route along which the triple-FOV HSRL was deployed during the 2020 Joint ECS and SCS cruise. (A) General diagram. (B) The selected part (the L1-L5 regions) for further investigation.


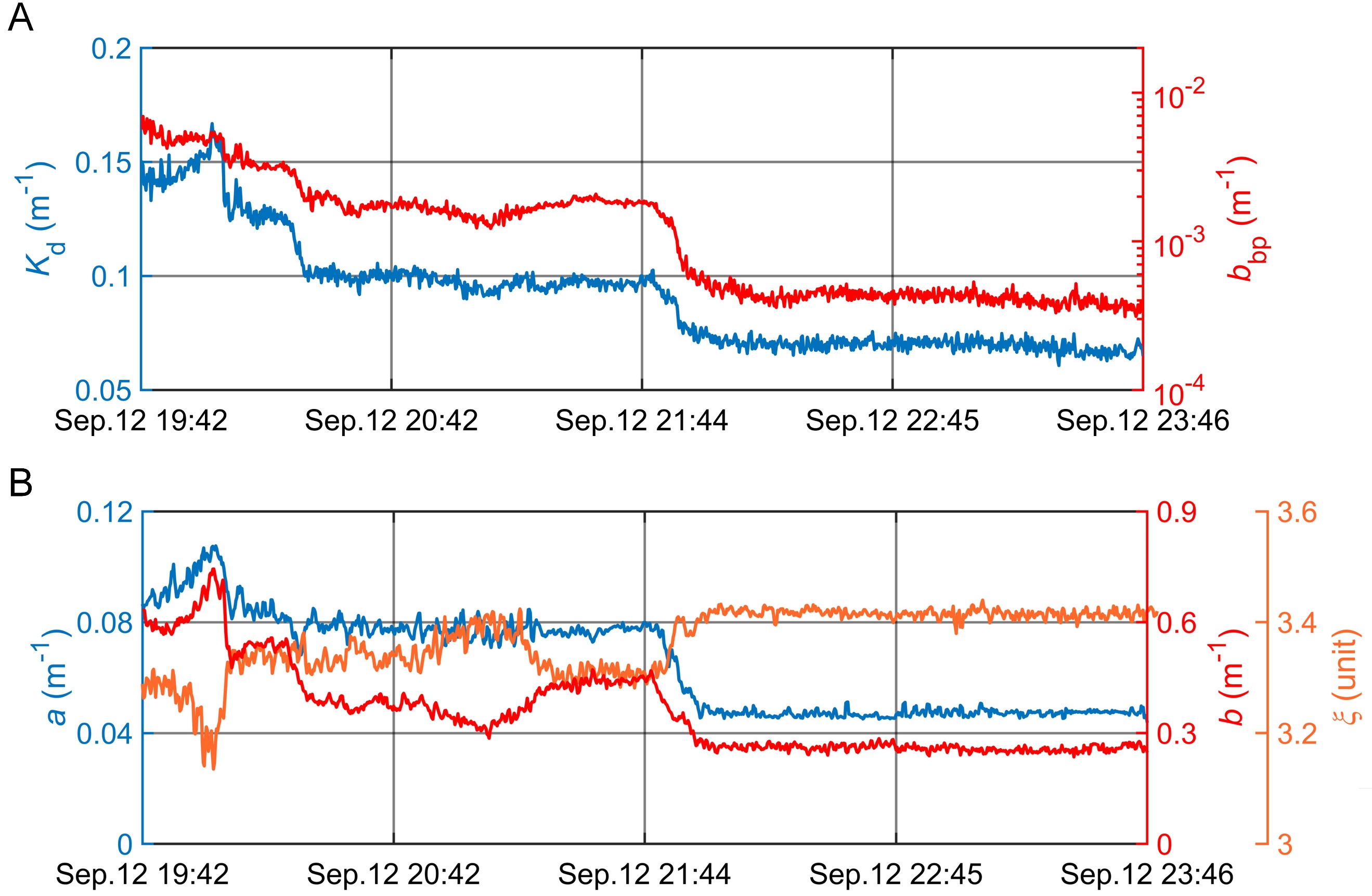


**Supplementary Fig. S4.** Column mean values of (A) diffuse attenuation coefficient *K*_d_ and particulate backscattering coefficient *b*_bp_, (B) absorption coefficient *a*, scattering coefficient *b*, and slope of PSD *ξ* in the selected area on Sep.12, 2020.


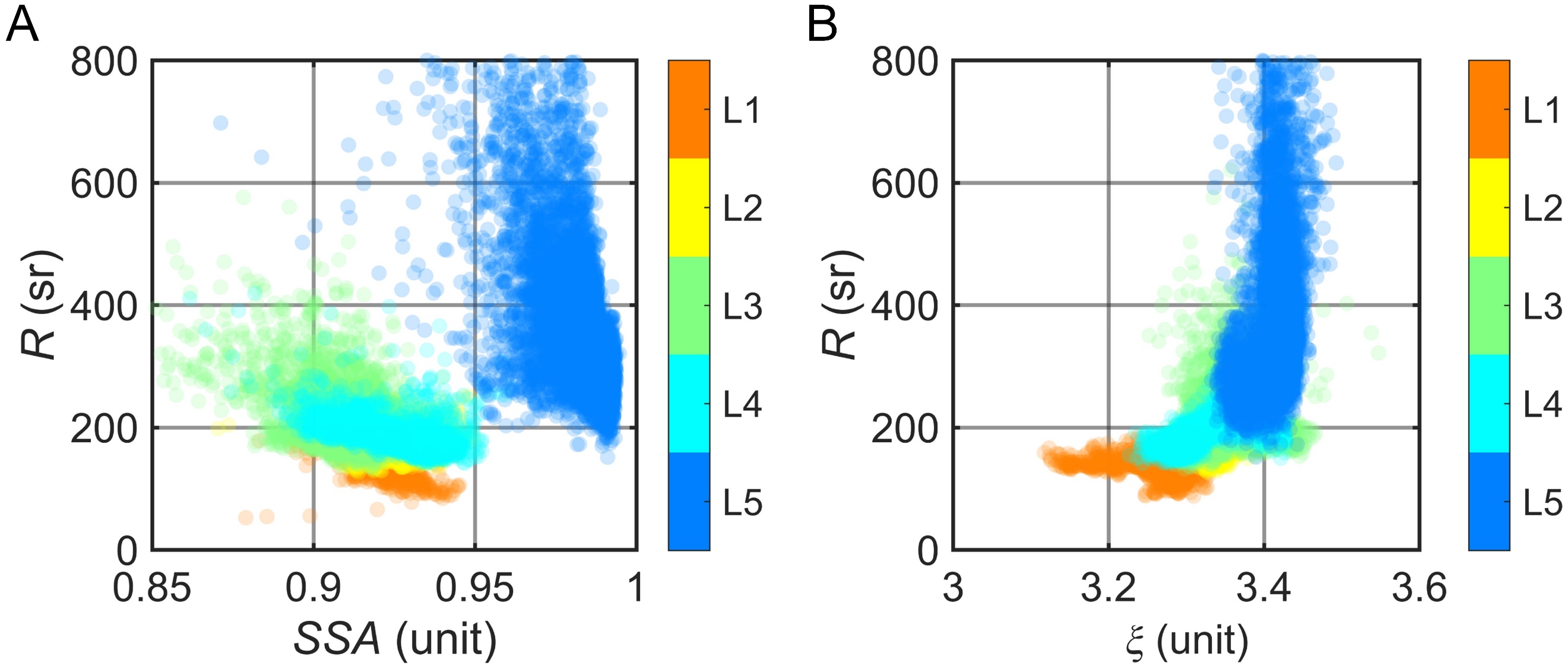


**Supplementary Fig. S5.** Clustering of seawater characteristics. (A) Lidar ratio *R* vs. single scattering albedo *ω*_0_. (B) Lidar ratio *R* vs. slope of PSD *ξ*.


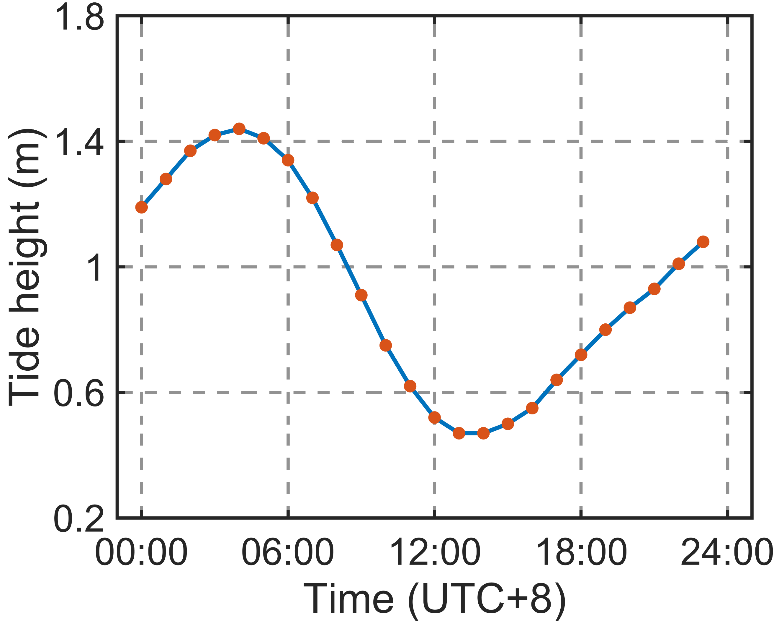


**Supplementary Fig. S6.** Hourly variation of tide height collected near the L4 region at Boao Harbor (20200912, 110.59˚E, 19.15˚N).


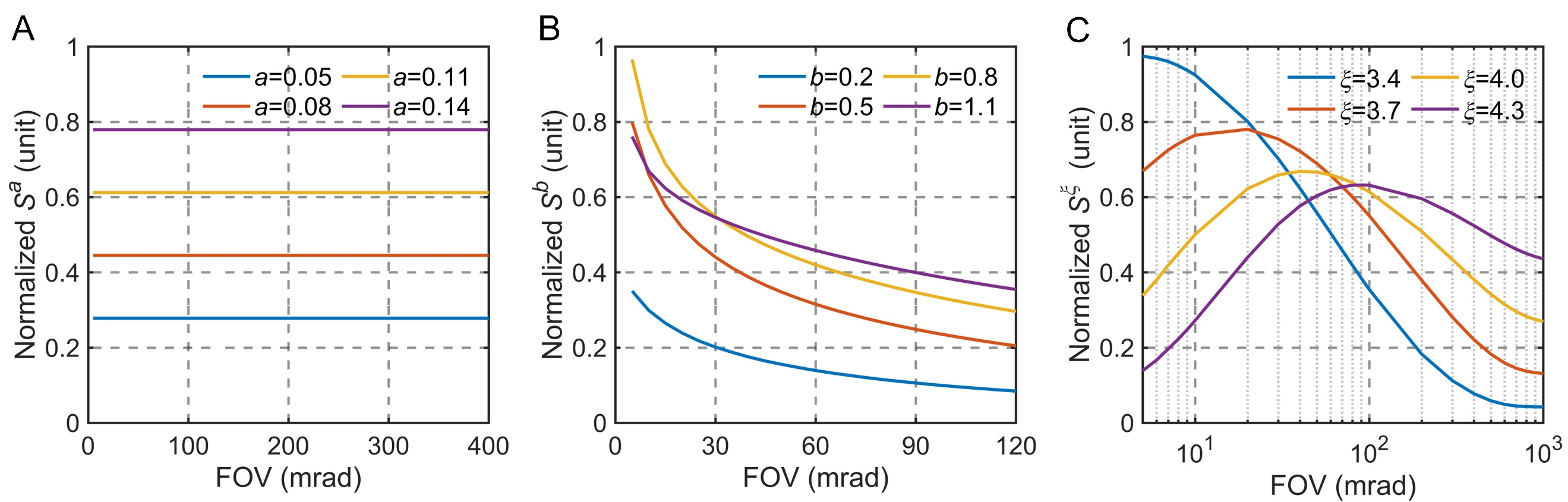


**Supplementary Fig. S7.** Signal sensitivities at 10 meter depth to (A) absorption coefficient *a*, (B) scattering coefficient *b*, and (C) slope of PSD *ξ*. When signal sensitivities to the other two variables are analyzed, *a*, *b*, *and ξ* are fixed to 0.08 m^-1^, 0.5 m^-1^, and 3.7, respectively.


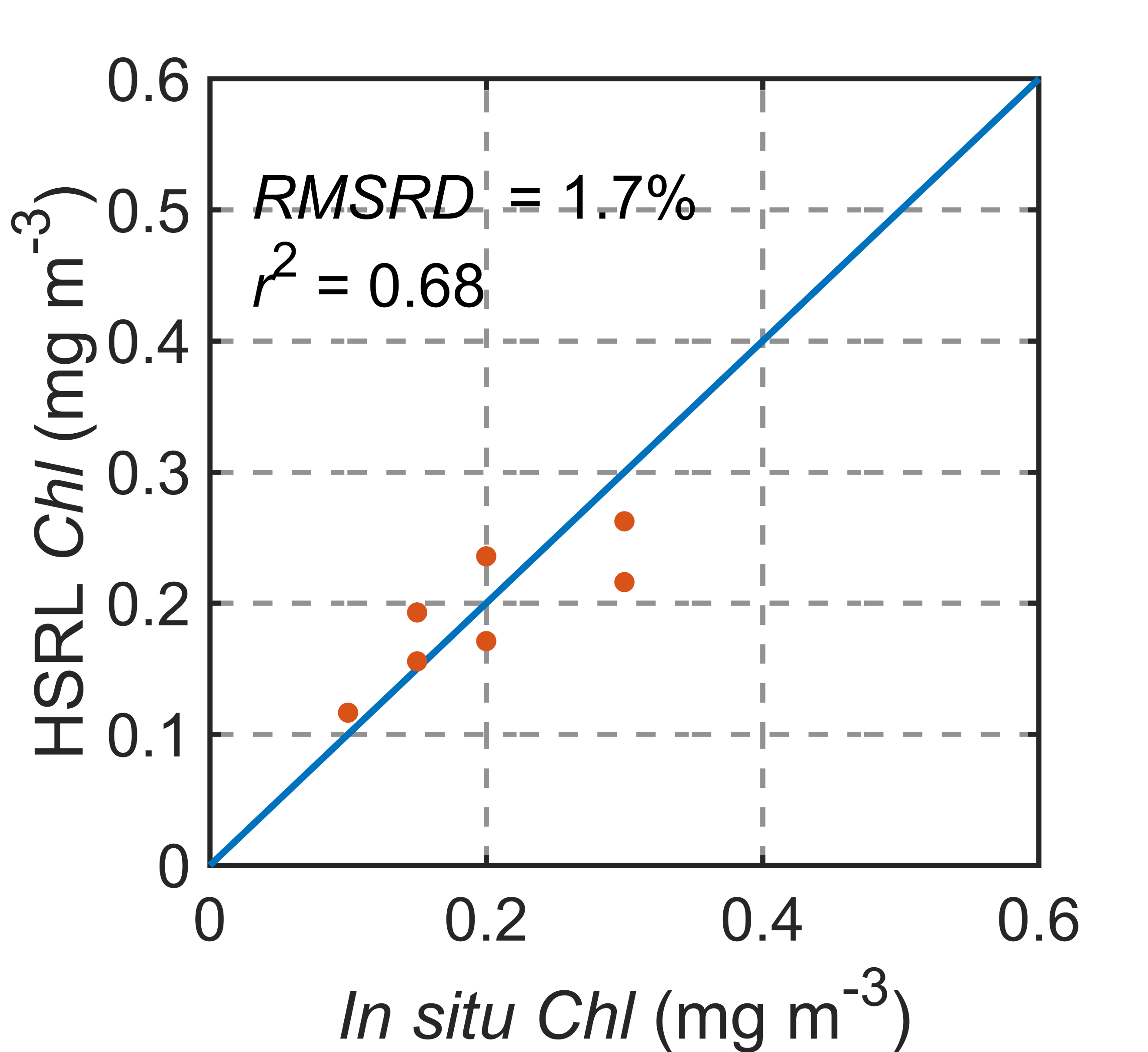


**Supplementary Fig. S8.** Comparison of *Chl* derived by *K*_d_ from the triple-FOV HSRL and *Chl* from *in situ* samplings.

# **Supplementary Tables**

# **Supplementary Table S1.** Parameterisation of TFOV-HSRL in MC simulations (others is same with **Supplementary Table S3**). The inclination angle is the angle between the optical axis and the horizontal plane.

| **Parameter** | **Value** | **Unit** |
| --- | --- | --- |
| Signal depth resolution | 0.3 | m |
| Height above the sea surface | 6.1 | m |
| Inclination angle | 30 | ° |
| Seawater refractive index | 1.34 | - |

**Supplementary Table S2.** Input *a*, *b*, and *ξ* in MC simulations.

| **Case No.** | ***a* (m^-1^)** | ***b* (m^-1^)** | ***ξ* (unit)** |
| --- | --- | --- | --- |
| 1 | 0.04896 | 0.01442 | 4.01159 |
| 2 | 0.04980 | 0.02298 | 3.87755 |
| 3 | 0.05050 | 0.03055 | 3.80840 |
| 4 | 0.05112 | 0.03754 | 3.76363 |
| 5 | 0.05169 | 0.04413 | 3.73133 |
| 6 | 0.05222 | 0.05041 | 3.70648 |
| 7 | 0.05272 | 0.05645 | 3.68653 |
| 8 | 0.05320 | 0.06230 | 3.67001 |
| 9 | 0.05365 | 0.06797 | 3.65601 |
| 10 | 0.05409 | 0.07350 | 3.64393 |
| 11 | 0.05785 | 0.12345 | 3.57426 |
| 12 | 0.06098 | 0.16761 | 3.54051 |
| 13 | 0.06376 | 0.20839 | 3.51925 |
| 14 | 0.06630 | 0.24683 | 3.50410 |
| 15 | 0.06867 | 0.28349 | 3.49251 |
| 16 | 0.07090 | 0.31875 | 3.48322 |
| 17 | 0.07303 | 0.35284 | 3.47552 |
| 18 | 0.07506 | 0.38595 | 3.46897 |
| 19 | 0.07702 | 0.41820 | 3.46329 |
| 20 | 0.09383 | 0.70963 | 3.42952 |
| 21 | 0.10781 | 0.96729 | 3.41194 |
| 22 | 0.12021 | 1.20522 | 3.40010 |
| 23 | 0.13156 | 1.42946 | 3.39119 |
| 24 | 0.14214 | 1.64338 | 3.38402 |
| 25 | 0.15210 | 1.84908 | 3.37802 |
| 26 | 0.16161 | 2.04799 | 3.37284 |
| 27 | 0.17069 | 2.24114 | 3.36829 |
| 28 | 0.17943 | 2.42933 | 3.36422 |

**Supplementary Table S3.** Main parameters of oceanic triple-FOV-HSRL.

|  | **Parameter** | **Value** | **Unit** |
| --- | --- | --- | --- |
| Transmitter | Wavelength | 532 | nm |
|  | Spectral bandwidth | 75 | MHz |
|  | Pulse energy | 10 | mJ |
|  | Pulse width | 10 | ns |
|  | Repetition frequency | 10 | Hz |
|  | Beam divergence | 1 | mrad |
| Receiver | Iodine filter absorption line | 1104 | - |
|  | Bandwidth of the filter | 3 | nm |
|  | Maximum acceptance angle of the filter | 10 | ° |
|  | Aperture diameter | 50.8 | mm |
|  | Narrow field of view | 40 | mrad |
|  | Middle field of view | 80 | mrad |
|  | Wide field of view | 200 | mrad |
|  | Sample rate | 400 | MSa/s |

# **Supplementary Table S4.** The measured data for the calibration of the triple-FOV. Three different groups (No. 1-3) of the edge points are recorded for each FOV.

| *d* = 5.481 m | **WFOV** | | | **MFOV** | | | **NFOV** | | |
| --- | --- | --- | --- | --- | --- | --- | --- | --- | --- |
| No. | 1 | 2 | 3 | 1 | 2 | 3 | 1 | 2 | 3 |
| *l_1_* (m) | 0.385 | 0.583 | 0.510 | 0.452 | 0.375 | 0.368 | 0.218 | 0.220 | 0.191 |
| *l_2_* (m) | 0.447 | 0.192 | 0.243 | 0.464 | 0.295 | 0.414 | 0.212 | 0.218 | 0.148 |
| *l_3_* (m) | 0.775 | 0.414 | 0.710 | 0.222 | 0.420 | 0.418 | 0.205 | 0.186 | 0.226 |
| Width (mrad) | 196.8±9.5 | | | 83.2±4.0 | | | 43.4±1.6 | | |

# **Supplementary references**

[1] Kostadinov TS, Siegel DA, Maritorena S. Global variability of phytoplankton functional types from space: Assessment via the particle size distribution. Biogeosciences, 2010, 7: 3239-3257
